# Macroscopic Hematuria due to Placenta Percreta: Report of Two Cases and Short Review

**DOI:** 10.1155/2017/9863792

**Published:** 2017-06-18

**Authors:** Ourania Koukoura, George Lialios, Antonios Garas, George Sveronis, Asterios Nidimos, Irondiana Gkorezi, Zoi Alevra, Vassilios Tzortzis, Athanasios Oeconomou, Ioannis Zachos, Alexandros Daponte

**Affiliations:** ^1^Department of Obstetrics and Gynecology, University Hospital of Larissa, Larissa, Thessaly, Greece; ^2^Department of Urology, University Hospital of Larissa, Larissa, Thessaly, Greece

## Abstract

Herein we present two cases of pregnant women with placenta percreta and severe hematuria during the 24th and 35th weeks of pregnancy, respectively. A timely sonographic diagnosis was feasible in the first case and cesarean section was performed during the 29th week. During the operation, the placenta was invading the bladder wall and concomitant hysterectomy with cystotomy and bladder wall reconstruction was performed. The second case presented in our emergency department with vaginal bleeding during the 35th weeks of pregnancy. She underwent an emergency cesarean section with uterine preservation, cystotomy, and bladder reconstruction.

## 1. Introduction

Placenta percreta represents a rare pregnancy complication where the placenta invades the uterine myometrium and occasionally adjacent organs, such as the bladder. The prevalence of all types of abnormally invasive placenta (accreta, increta, and percreta) has been increased due to the rise in the cesarean section rate. It is estimated that invasive placenta cases have risen to 10 times over the last 50 years. Approximately 1/500 to 1/2500 deliveries are complicated by invasive placenta [1]. Placenta percreta is associated with significant morbidity. When placenta percreta is invading the bladder, mortality rates may be as high as 9.5% and 24% for mother and baby, respectively [2]. Herein we report two cases of placenta percreta with bladder invasion, one of which was diagnosed prenatally during the second trimester of pregnancy. In the second case, the diagnosis was made antenatally during the 35th weeks of pregnancy immediately prior to the emergency cesarean section.

## 2. Case Presentation

### 2.1. Case  1

**Figure 1:**
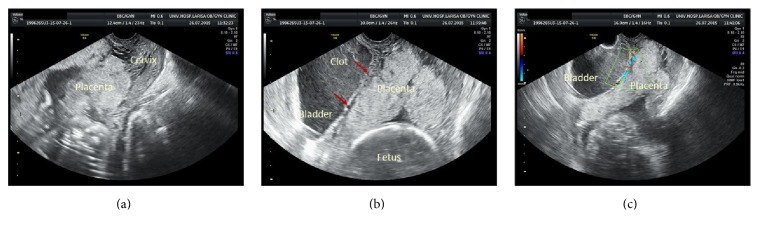
(a) Complete placenta previa. (b) Disruption of the bladder-placental interface indicated by red arrows. (c) Color Doppler revealed hypervascularity between the placenta and the bladder.

A 39-year-old woman (gravida 3, para 2) was referred to our department during the 24th week of pregnancy due to gross hematuria. Τhe pregnancy was uncomplicated till then; however the diagnosis of complete placenta previa was made during the anatomic scan three weeks prior to her admission. She had two previous cesarean sections and no other medical history. An abdominal sonographic evaluation revealed a viable fetus with appropriate biometrical parameters and normal amniotic fluid, while transvaginal scan suggested the presence of complete placenta previa with bladder wall invasion (Figure 1). She was admitted to our department for expectant management. A course of steroids was administered to promote fetal lung maturation. On hospital day #1, bladder irrigation was performed by the urologists and a Foley catheter was placed in order to control hematuria. The patient had intermittent macroscopic hematuria and cystoscopy was performed on day #4 in order to confirm the diagnosis; however the examination was inconclusive. On hospital day #10, evaluation of the placenta with magnetic resonance imaging (MRI) was also nondiagnostic. The patient was asymptomatic by day #20 and she was discharged from the hospital. Two days later, however, the woman became symptomatic and was then readmitted with hematuria and vaginal bleeding. She had a hematocrit of Ht 22.1% and hemoglobin Hb 6.8 mg/dL and was transfused with two units of blood. The patient went into labor and she was immediately transferred to the operating room. A multidisciplinary team by obstetricians, midwifes, urologists, neonatologists, and anesthetists was on site. A viable male baby, weighing 1000 gr, was delivered by cesarean section and concomitant abdominal hysterectomy. The baby was transferred to the NICU where he was intubated. Dissection of the placenta from the anterior uterine wall created a large defect on the dome of the bladder. The bladder was repaired with two layers of continuous Vicryl 0 suture. A Foley catheter and an intraperitoneal drain were placed. The patient required transfusion of 20 units of various blood products intraoperatively. The postoperative course was uneventful and the patient was discharged on the 10th postoperative day. She made a good recovery; however urologic evaluation one month postoperatively revealed a vesicovaginal fistula, which was surgically treated at a later date. The baby was discharged three months after delivery.

### 2.2. Case  2

A 26-year-old woman (gravida 4, para 3) presented in our emergency department with vaginal bleeding during the 35th week of pregnancy. She had three previous cesarean sections and no antenatal screening during the present pregnancy. She was transferred to the labor ward where appropriate for gestational age embryo and anterior placenta previa detected during abdominal ultrasound scan. An emergency cesarean section was scheduled and just prior to the operation a Foley catheter was inserted to the bladder which drained blood. The cesarean section was carried out with a vertical incision high in the anterior uterine wall. A male baby weighing 2250 gr was delivered with Apgar scores of 8 and 9 and was transferred to the NICU for further evaluation. Interrupted Vicryl 1 sutures were used for closure of the uterine wall. Intraoperative bleeding was moderate and no transfusion was necessary at that point. A urologist evaluated and repaired the bladder defect which was away from both ureteral orifices. The patient made an unremarkable recovery and both she and the baby were discharged on the 5th postoperative day. The Foley catheter was removed on the 10th postoperative day.

## 3. Discussion

The exact incidence of placenta percreta remains unclear, since the invasiveness of the placenta is not always established, making it difficult to distinguish between placenta percreta, increta, and accreta. Earlier reports describe the incidence of placenta percreta as within 1 : 1000 to 1 : 70,000 births [3]. Placenta percreta represents the most severe form of placental invasiveness which requires aggressive evaluation and management to decrease morbidity. Multiparity, prior uterine surgery, most commonly cesarean section, and advanced maternal age have been all identified as risk factors for developing this complication of pregnancy [4]. There is a linear increase in the incidence of invasive placenta with the number of previous cesarean deliveries. Diagnosis should always be suspected in any pregnancy with previous uterine scar and detection of a low-lying placenta or placenta previa. It is estimated that when the detection of placenta previa is made in a woman with three previous cesarean sections, there is a 40% chance of the occurrence of placenta accrete [5]. Both cases in our report had previous cesarean sections and, in the first case, the diagnosis of placenta previa was established during the 21st week's anatomic scan.

Prenatal diagnosis of placenta percreta can help reduce maternal/fetal morbidity and mortality by allowing us to choose the best time and place of delivery. Specific sonographic criteria have been recommended for a timely diagnosis of morbidly adherent placenta. Detailed medical history and high index of suspicion is essential in these cases. The sonographic features include an inability to visualize the normal retroplacental clear zone, irregularity of the serosa-bladder interface, intraplacental lacunar spaces, and hypervascularity between the placenta and the bladder when using the color Doppler, resembling a large aneurysm [6–9]. Transvaginal scan was indicative of placental invasion in our first case, since there was a distinct disruption of the bladder wall (Figure 1(b)). We employ the use of magnetic resonance imaging (MRI) to confirm diagnosis. Magnetic resonance imaging (MRI) has similar performance status to the ultrasound; however it can be helpful in cases where placenta is difficult to visualize on ultrasound due to the patient's high body mass index. Expertise in such cases is often required to obtain an accurate diagnosis [10]. Although sonographic evaluation revealed a high suspicion of placental invasion in our first case, MRI was nondiagnostic and final diagnosis of bladder invasion was confirmed intraoperatively.

Hematuria is a rare occurrence comprising 25% of all placenta percreta cases [11]. Although not a sensitive finding, placenta percreta with bladder invasion should be suspected in any pregnant woman presenting with gross hematuria and a history of previous cesarean sections [12]. Placental bladder infiltration is a potentially life-threatening situation and therefore mandates a multidisciplinary surgical management, adequate number of blood products, neonatal intensive care, and the option of uterine artery embolization if needed. Hysterectomy without any attempts to remove the placenta is the recommended option [13]. Separation of the bladder from the placenta may cause severe blood loss. Even in cases with major intraoperative bleeding, bladder integrity preservation should be one of the primary surgical goals. Cesarean section and hysterotomy can be carried out through the bladder incision whenever this is unavoidable. Preoperative placement of ureteric stents is essential to minimize ureteral injury.

Another more recent optional management involves the placenta to be left in situ during cesarean delivery and postoperative methotrexate injection [14, 15]. Concomitant vascular embolization minimizes intraoperative blood loss and may facilitate postoperative placental involution. In such cases, regular postpartum monitoring is required, since complete regression of the placental remnants may take several months to ensue. None of our cases were treated conservatively. Although diagnosis in the first of our cases was made several weeks prior to the operation, emergency cesarean section and hysterectomy were performed based on the sudden deterioration of the patient's condition. In the second case, cesarean section was performed with a high vertical incision and no attempts to separate the bladder were made. Blood loss was controllable and hence hysterectomy was avoided [16]. Bladder was repaired with two layers of sutures in both cases. Potential long-term consequences of bladder damage include fistulas, urinary incontinence, and sexual dysfunction.

Patients with suspected placenta percreta with bladder invasion should be counseled about the potential risks of all management options. The surgical plan should be documented and alternative treatments should be available. The woman should become aware that efforts to preserve her uterus might increase morbidity and that planned hysterectomy is a safer option than peripartum hysterectomy.
